# 

**Published:** 1822-09

**Authors:** 


### LECTURES ANNOUNCED.

Dr. Richard Harrison, on Physiological Pathology and the Practice of Physic; and Dr. Paris, on Pharmaceutical Chemistry, with Materia Medica, including Pharmacology and the Doctrines of Therapeutics.

Dr. Gregory, on the Theory and Practice of Physic, and the Materia Medica.

Mr. C. Bell and Mr. Shaw, on Anatomy and Surgery, and Dissections, with Demonstrations.

Dr. Southey, on the Theory and Practice of Physic; Mr. Wood, on Chemistry; Dr. Merriman and Dr. Ley, on Midwifery and Diseases of Women, &c.

Dr. Clutterbuck, on the Theory and Practice of Physic, Materia Medicine, and Chemistry.

Dr. Pearson, on the Laws of the Animal Economy and the Practice of Physic, with Pathological Demonstrations; and Therapeutics, with Materia Medica.

W. T. Brande, Professor of Chemistry, on the Science of Chemistry.

Dr. Davis, on Midwifery, and the Diseases of Women and Children.

Dr. Uwins, on the Theory and Practice of Medicine.

Mr. J. Harrison Curtis, on the Anatomy, Physiology, and Diseases of the Ear, and on the Medical Treatment of the Deaf and Dumb.

Mr. Brookes, on Anatomy, Physiology, and Surgery.

Mr. Grainger, on Anatomy and Physiology; Dr. Armstrong, on the Principles and Practice of Physic; Dr. Elliotson and Mr. Phillips, on Materia Medica, Chemistry, and Pharmacy; Dr. Elliotson, on Human and Comparative Physiology and Forensic Medicine.

Dr. Hue, on Medicine, Chemistry, and Materia Medica; Mr. Abernethy, on Anatomy, Physiology, and Surgery; Dr. Gooch and Dr. Conquest on Midwifery; Mr. Stanley, on Practical Anatomy, with Demonstrations.

The Winter Course of Lectures at the Medical School, London Hospital.

Mr. Blagden and Mr. Stone, on Midwifery and the Diseases of Women, &c.

Dr. Henry Davies, on Midwifery and the Diseases of Women and Children.

---

### MONTHLY CATALOGUE OF MEDICAL BOOKS.

Popular Directions, collected from Experience, for the Prevention and Cure of Head-Aches, Colds, and Indigestion, with Medical Prescriptions and Cases interspersed; with the most useful Remarks on those Subjects in the Works of Abernethy, Hamilton, Cooper, and Wilson Philip. By an experienced Medical Practitioner. 18mo. 2s. 6d.

An Appendix to a Catalogue of Books in Anatomy, Medicine, Surgery, Midwifery, Chemistry, Botany, &c. containing a complete List of Books on the above Sciences, published since October 1821; with all the useful and approved Class Books on sale at Anderson's Medical Library, 40, West Smithfield.

Synopsis Nosologiæ Methodicæ exhibens Systema Nosologicum, Auctore Gulielmo Cullen, M.D. &c. Editio altera. 32mo. 2s.

Gregory on the Duties and Qualifications of a Physician, more particularly addressed to Students and Junior Practitioners. A new Edition. 12mo. 4s.

---

### NOTICES TO CORRESPONDENTS.

*We have received a variety of interesting Communications, which will be published as nearly as possible in the order of their dates.*

C. P. *will observe that we have anticipated his suggestion, having already devoted the* Collectanea *to a Selection of the most important Communications from other Works.—Lest we should be expected to adopt every hint with which our Correspondents may favour us, we would just remind them of the fable of the Old Man and his Ass.—If* C. P. *will send his address to the Publisher, the volumes of the Journal which he wants shall be forwarded to him, or he may procure them by giving an order to any of the Newcastle Booksellers. In either case, the price will be fifteen shillings each volume.*

*Some Communications of a nature entirely personal have been received: we have already declared our intention of declining to publish any such; and we trust that the good sense of the writers in question will convince them of the propriety of abiding by our resolution. If any of these Gentlemen will follow the example of one of our Correspondents in the present Number, by sending us any information of a professional nature, they shall not have any reason to complain of our backwardness in giving it to the public.*

